# The Role of Response Elements Organization in Transcription Factor Selectivity: The IFN-β Enhanceosome Example

**DOI:** 10.1371/journal.pcbi.1002077

**Published:** 2011-06-16

**Authors:** Yongping Pan, Ruth Nussinov

**Affiliations:** 1 Basic Science Program, SAIC-Frederick, Center for Cancer Research Nanobiology Program, NCI-Frederick, Frederick, Maryland, United States of America; 2 Sackler Institute of Molecular Medicine, Department of Human Genetics and Molecular Medicine, Sackler School of Medicine, Tel Aviv University, Tel Aviv, Israel; University of Illinois, United States of America

## Abstract

What is the mechanism through which transcription factors (TFs) assemble specifically along the enhancer DNA? The IFN-β enhanceosome provides a good model system: it is small; its components' crystal structures are available; and there are biochemical and cellular data. In the IFN-β enhanceosome, there are few protein-protein interactions even though consecutive DNA response elements (REs) overlap. Our molecular dynamics (MD) simulations on different motif combinations from the enhanceosome illustrate that *cooperativity* is achieved via unique organization of the REs: specific binding of one TF can enhance the binding of another TF to a neighboring RE and restrict others, through overlap of REs; the order of the REs can determine which complexes will form; and the alternation of consensus and non-consensus REs can regulate binding specificity by optimizing the interactions among partners. Our observations offer an explanation of how specificity and cooperativity can be attained despite the limited interactions between neighboring TFs on the enhancer DNA. To date, when addressing selective TF binding, attention has largely focused on RE sequences. Yet, the order of the REs on the DNA and the length of the spacers between them can be a key factor in specific combinatorial assembly of the TFs on the enhancer and thus in function. Our results emphasize cooperativity via RE binding sites organization.

## Author Summary

An enhanceosome is a functional unit that consists of DNA segment called enhancer; its transcription factors (TFs); and their interacting cofactors. To function, the TFs must assemble on their corresponding response elements (REs) cooperatively. Understanding how TFs assemble is important because the TF combination on the enhancer spells gene-specific activation (or repression). Traditional studies focused mainly on the derivation of consensus DNA sequences, and the TF interaction with its respective RE. This yielded limited success in deciphering the mechanism of selective TF binding. Here, in addition to the conventional roles of protein and DNA, we studied the organization of REs. The IFN-β enhanceosome is a good example because there are limited protein-protein interactions between consecutive TFs. Our molecular dynamics simulations revealed that cooperativity is achieved via overlap of REs, in addition to sparse protein-protein interactions. That is, because the REs overlap, binding of neighboring TFs affect each other through DNA conformation perturbation. In addition, alternation of consensus and non-consensus REs along the enhancer allows more efficient binding of TFs, while the order of the REs excludes unwanted TFs, and enhances selective TF binding. Our findings emphasize the overlooked role of the order and organization of REs, and the length of spacers between consecutive REs.

## Introduction

Cellular response to environmental signals relies on tight gene regulation. Specific recognition of response elements (REs) by transcription factors (TFs) [1]–[4] and their combinatorial assembly [1], [5], [6] on promoters and enhancers is crucial for functional, gene-specific transcription initiation [7]. However, *how* TFs recognize specific REs along the genome which contains hundreds of thousands of similar RE sequences, *how* the TFs and their co-regulators assemble to form the enhanceosome which is the functional unit, and *how* the RE organization on the enhancer DNA (the order of the REs on the DNA stretch and the spacer sizes between consecutive REs) play a role in the specificity are still open questions. It has been argued that the cell is populated by a large number of copies of the TF [1], [4], [8]. Consequently, all chromatin-exposed REs will be bound by their corresponding TF, *if* the TF can be favorably accommodated on the enhanceosome [1], [6], out-competing other TFs. Conformational ensembles of the RE-bound TFs will undergo allosteric, DNA-induced population shifts, which would alter the TFs' co-factor binding sites to binding-favored states [1], [9]. Whether the RE-bound TF will affect function depends on factors such as co-factor availability and post-translational modification state, which relate to the cellular environment. RE availability is governed by chromatin packaging and re-modeling [10], which is determined by the organism's developmental state and cellular environment.

Selective RE recognition and TF activation on chromatin-exposed DNA were proposed to reflect three factors [1]: (i) the cellular network (or environment) which determines the post-translational modification states, co-factor concentration, etc; (ii) protein and DNA which exist as dynamic conformational ensembles that re-distribute allosterically upon binding, post-translational modification, external conditions, etc; and (iii) tight packing of multiple TFs and co-regulators in enhanceosomes (or promoters). This last factor relates to TFs shapes and sizes, and lengths of intervening DNA stretches between neighboring REs [1]. Although dubbed in the literature as ‘combinatorial assembly’, the implications as specificity-determining factor in RE recognition have largely been overlooked.

Enhanceosomes often involve tens of TFs [1], [2], [11] packed along a DNA stretch of several hundreds of bps [1], [2], [12], [13]. REs typically occur in clusters with spacers of variable lengths where REs can also overlap [6], [14]. Given the large number of possible REs, and RE nucleotide sequence redundancy, the question of how specific TFs prevail over others for given REs is crucial since each RE is associated with a different gene and thus a different function [1], [2], [9].

**Figure 1:**
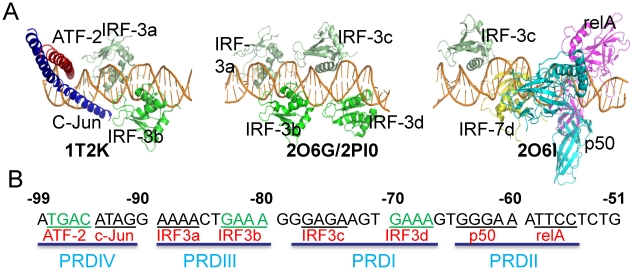
Structural information of the IFN-β enhanceosome. (A) The four crystal structures used in the simulations. The PDB codes and the protein components are labeled. The three structures are rendered in a same orientation with respect to the DNA sites. The full lengths of DNAs in each of the structures are not shown for clarity. The structure of 2PI0, which is very similar to that of 2O6G, is not shown. (B) The DNA sequence of the IFN-β enhancer, which was divided into four positive regulatory domains PRDIV, III, I and II from upstream to downstream. Core binding site for each protein is underlined separately. Consensus binding sites are in green. Base positions are labeled with respect to the transcription initiation site.

The IFN-β enhanceosome has been a model system for transcription regulation due to its small size. While a typical enhanceosome functions through long-range interactions [15], the IFN-β enhanceosome sits only tens of bps upstream of the IFN-β gene transcription initiation site and recruits co-factors such as p300 [16] which acetylates histone H1 [17]. The acetylation of histone ‘loosens’ the nucleosomes at the TATA box region, exposing the promoter, thus promoting assembly of the general transcription factor TFIIB and RNA polymerase II [18] which leads to transcription initiation [8]. IFN-β gene expression requires a minimal number of 8 proteins on the enhancer (Figure 1): ATF-2/c-Jun dimer, four IRF-3 and/or IRF-7 proteins, and an NFκB dimer (typically p50 and p65) [19], that are activated through three different pathways [20]–[22]. The synergistic [23], thus orderly [8] assembly is assisted by the HMG I (Y) protein [24], [25]. Once the IFN-β protein is expressed to a certain level, it dramatically increases IRF-7 expression, which further promotes the re-assembly of the enhanceosome with the IRF-7 incorporated [26].

The IFN-β enhancer is composed of four positive regulatory domains (PRDs), IV, III, I, and II from positions −99 to −55 with respect to the transcription initiation site (Figure 1). Several crystal structures are available [27]–[30], each of which encompasses part of the enhanceosome (Figure 1). p50 has been shown to bind to the IFN-β enhancer prior to viral entry, while completion of the assembly of all 8 TFs on the DNA occurs after infection [31]. Of interest, binding of IRF-3 at PRDIII depends on the ATF-2/c-Jun heterodimer orientation on the DNA [32]. PRDIV is composed of two components, the consensus for ATF-2 binding and non-consensus for c-Jun (Figure 1); similarly, PRDII is also divided into two non-symmetric parts: the 5′ site is recognized by p50 and the 3′ site by RelA [33]. The four IRF-3 binding sites within PRDI and PRDIII are also arranged in alternative consensus and non-consensus motifs (Figure 1). Crystal structures of the DNA/IRF-3/IRF-7 complex indicated that IRF-3 binds site C (and/or A) and IRF-7D (and/or B). Understanding how these loosely packed TFs communicate with each other and the role of the REs organization in TF selectivity is important for deciphering the mechanism of cooperative assembly. Using MD simulations and modeling we show that despite the sparseness of protein-protein interactions within the enhanceosome, packing along the DNA is already maximized: binding of each of the four enhanceosome TF dimers to their respective REs cooperatively influences the association of a neighboring pair, by partially pre-configuring the overlapped segment of the neighboring binding sites. We also show that the arrangement of consensus and non-consensus binding sites on the DNA facilitates the optimization of the binding of TF partners. The emerging picture from our results is that overlap of REs leads to specificity by enhancing binding of one TF and restricting others. Together, our results can provide an explanation for how specific assembly on enhancer DNA can be achieved despite the limited protein-protein interactions within the assembly.

## Results

To gain insight into TFs-REs binding selectivity and the role of the REs organization on enhancer DNA, MD simulations and structural analysis were performed on complexes derived from three crystal structures (Figure 1) of the virus-inducible IFN-β gene enhanceosome. These structures are incomplete entities of the enhanceosome. A striking structural feature of this enhanceosome is the sparseness of interactions among the proteins which to date has not been observed for other systems. Figure 1 in Text S1 shows all interactions within 4.5 Å between the proteins. Since nonetheless information has to be communicated among the TFs, we focus on potential allosteric conformational changes in the DNA upon protein binding within and outside the binding sites. In addition, because IRF-7 prefers sites B and D while IRF-3 prefers A and C, we closely monitored their interaction energy differences. Using interaction energy instead of binding free energy to assess the association is based on the assumption that the trend of interaction energy parallels that of the binding free energy. This generally holds for such systems since the DNA binding domains are fairly well structured; the binding motifs of ATF-2 and c-Jun and of IRF-3 and IRF-7 are very similar to each other; and the entropy term differences are often negligible. However, it should be kept in mind that these are large systems. While based on the structural fluctuation properties the MD simulation results were interpreted with the assumption that steady-state equilibrium was reached, it is possible that much longer simulations may reveal further dynamic changes not captured in this work.

### Dynamics of the 1t2k complex reveals high flexibility

**Figure 2:**
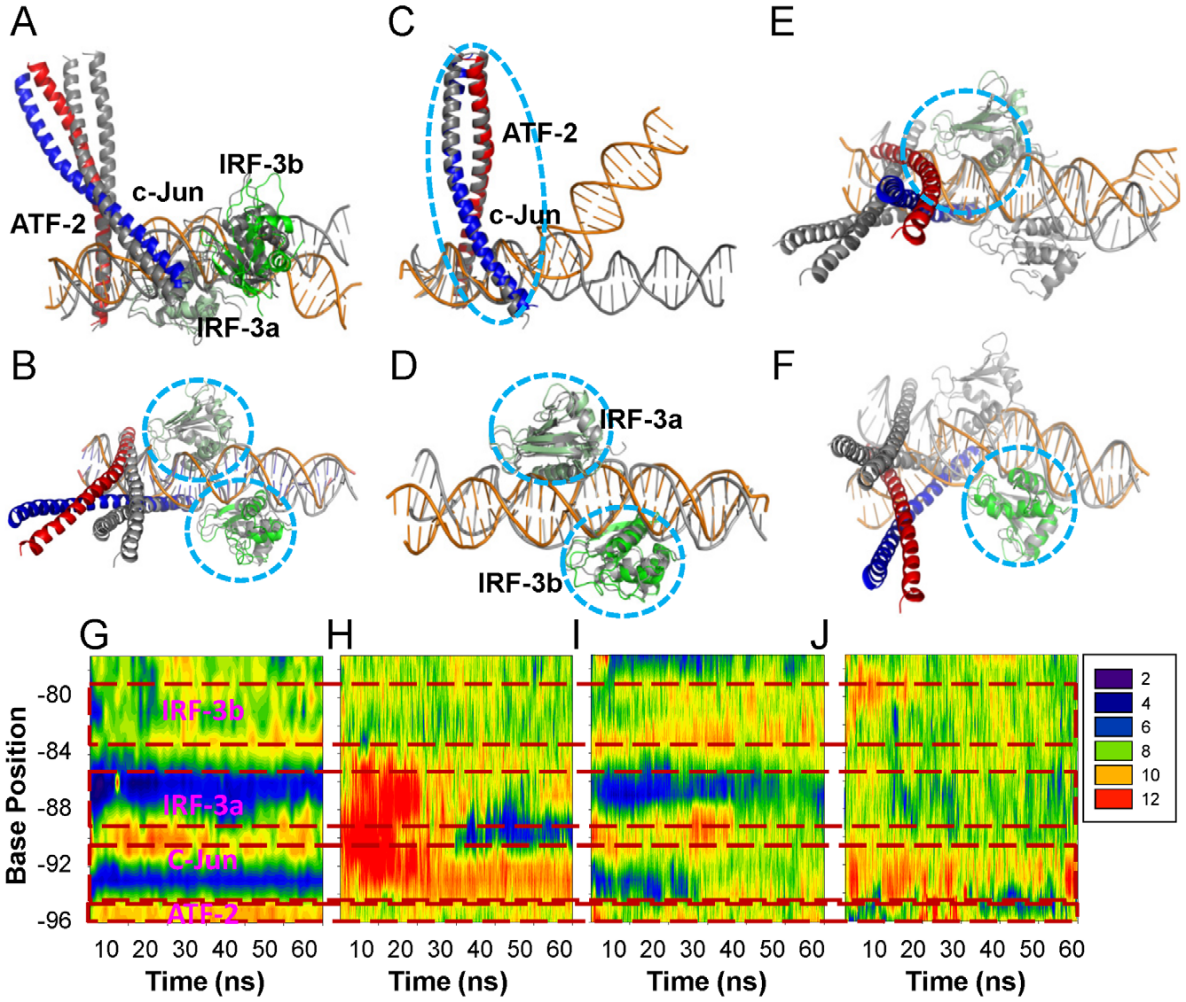
Conformational changes of various structures derived from 1T2K upon MD simulations. (A–F) Superposition of average structures over the last 10 ns with crystal conformation of 1t2k complex. (A and B) full complex; (C) DNA/ATF-2/c-Jun; (D) DNA/IRF-3a/IRF-3b complex; (E) DNA/ATF-2/c-Jun/IRF-a; and (F) DNA/ATF-2/c-Jun/IRF-3b complexes. The crystal conformation is in gray. Other coloring schemes are as in Figure 1. Structural motifs used for partial superimpositions are highlighted in circles. (G–J) DNA minor groove width dynamics during the 60 ns trajectory for simulations of the full complex, DNA/ATF-2/c-Jun, DNA/IRF-3a/IRF-3b and DNA, respectively. DNA regions that correspond to binding sites of proteins were boxed in red and labeled with protein names.

**Figure 3:**
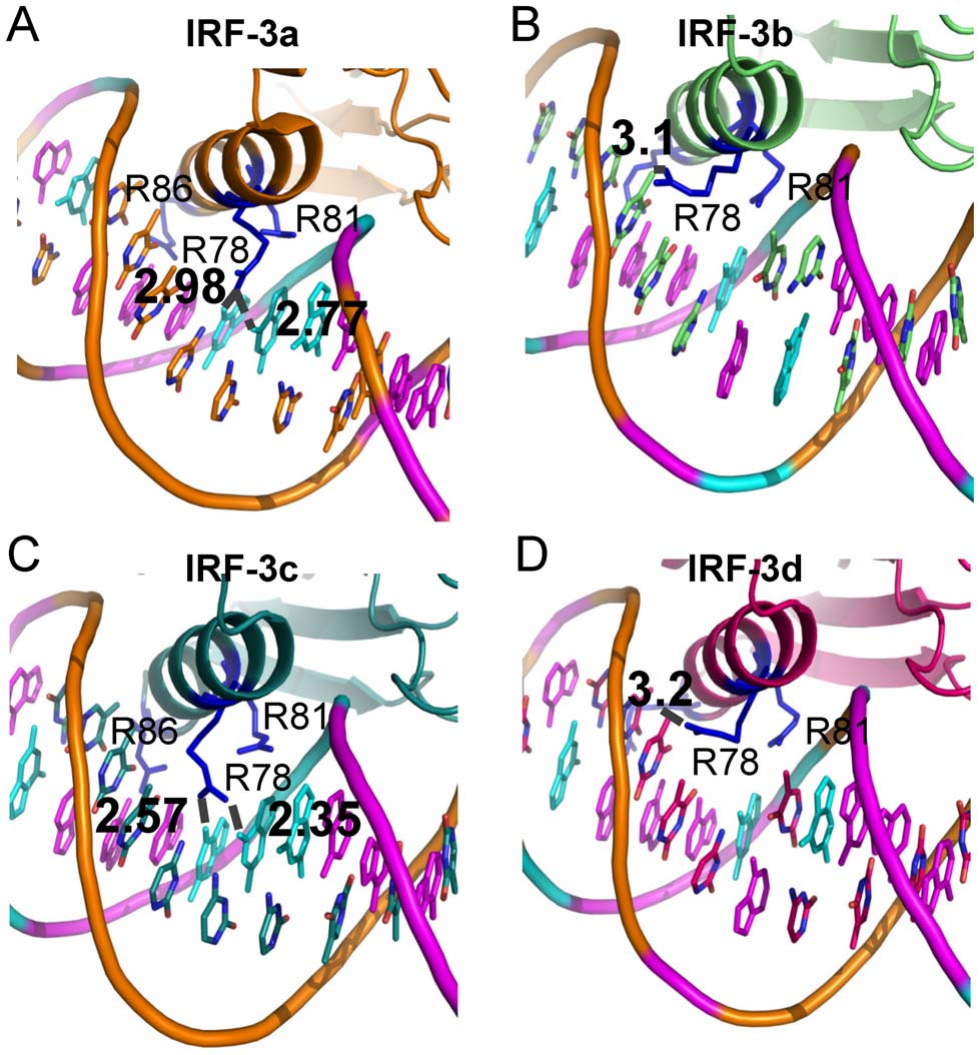
Binding pattern differences for residues Arg78, Arg82 and Arg86 in crystal structure 2O6G. (A), (B), (C) and (D) show conformations at binding sites IRF-3a, -3b, -3c and -3d, respectively. Bases G and A are shown in cyan and magenta, respectively while other bases are colored based on atom type. Arg78 binds similarly through HB interactions with 2 consecutive G bases in sites A and C while Arg78 pointed away to interact with a T base. Arg78 failed to form HB with the G base in sites B and D due to the presence of a T base next to the G base.

Molecular dynamics (MD) simulations were performed on various combinations of the structural motifs from the 1t2k crystal structure (Table 1). Figure 2 shows the conformational changes of each simulated system with average structures from the respective trajectories superimposed onto the crystal structure. Several observations were made: 1) the full complex was unexpectedly flexible, with the DNA deviating significantly from the crystal structure (Figure 2a). However, the local DNA conformations at the sites where the proteins were bound were relatively stable (Figure 2b); 2) when the two IRF-3 proteins were removed, the DNA bent toward the ATF-2/c-Jun motif with large magnitude, while the DNA conformation in the ATF-2/c-Jun region was reasonably retained (Figure 2c). When the ATF-2/c-Jun motif was removed, the DNA conformation deviated less from the crystal (Figure 2d); 3) when one IRF-3 was removed, the conformation of the DNA at that IRF-3 site drifted away while the IRF-3 bound region still conserved the crystal conformation (Figure s 2e, f). As expected, when simulated alone, the DNA relaxed and lost its unique conformational features such as kinks present in the crystal structure (Figure 2j), and the ATF-2/c-Jun hetero dimer demonstrated high flexibility during the 60-ns trajectory (data not shown). Further analysis showed that IRF-3A anchored well into the major groove throughout the trajectory while IRF-3B was ejected from the major groove to some extent (data not shown). This may have to do with binding specificity and tightness of each IRF molecule. IRF-3A binding was more specific (more hydrogen bonds (HBs) with bases) while IRF-3B was less so, as further discussed later. These results show that the overall complex is quite flexible due to the sparse protein-protein interactions, and in the absence of protein binding the DNA conformation easily deviates from the protein-bound crystal structure.

**Table 1:** List of all simulations.

1t2k	2o6g	2pi0	2o61
DNA/ATF2/cJun/IRF3a/IRF3b	DNA/IRF3a/IRF3b/IRF3c/IRF3d	DNA/IRF3a/IRF3b/IRF3c/IRF3d	DNA/IRF3c/IRF7d/p50/RelA
DNA/ATF2/cJun	DNA/IRF3a/IRF3b	DNA/IRF3a/IRF3b	DNA/IRF3c/IRF7d
DNA/IRF3a/IRF3b	DNA/IRF3c/IRF3d	DNA/IRF3c/IRF3d	DNA/p50/RelA
DNA/ATF2/cJun/IRF3a	DNA/IRF3a/IRF3c	DNA/IRF3a/IRF3c	DNA
DNA/ATF2/cJun/IRF3b	DNA/IRF7b/IRF7d	DNA/IRF7b/IRF7d	
DNA	DNA	DNA	

### Structural comparison and dynamic data reveal the importance of binding site order

Although it is expected that the DNA conformation will fluctuate due to the lack of significant interactions between the proteins, the extent of DNA bending in the DNA/ATF-2/c-Jun simulation was still surprising. Inspection of the crystal structure revealed that the DNA conformation at the c-Jun site appeared unusual as it had few contact with the c-Jun arm on the right hand-side (Figure 1, Figure 2a in Text S1). To quantitatively characterize the DNA conformation, we calculated groove parameters. Because the four DNA groove parameters are inter-correlated (larger major groove width corresponds to smaller major groove depth; smaller minor groove width to larger minor groove depth), table 2 presents only the minor groove depths. The largest are at -93T and -87A, where His40 and Leu42 from IRF-3A and IRF-3B interact with the minor groove. Comparison with a similar crystal structure illustrates that the uniqueness of this conformation (Figures 2a, b in Text S1) is due to the presence of IRF-3A. This explains the dramatic DNA conformational change in the DNA/ATF-2/c-Jun complex simulation, because upon removal of IRF-3A, the DNA/ATF-2/c-Jun motif had to adjust its conformation to optimize the interactions, resulting in large changes.

**Table 2:** DNA helical parameters for crystal structure 1t2k T_(-102)_ AA_(-100)_ ATGACATAGG_(-90)_ AAAACTGAAA _(-80)_ GGGAGAAG_(-72)_.

	-93T	-92A	-91G	-90G	-89A	-88A	-87A	-86A	-85C	-84T
Major width	**13.9**	**14.6**	12.0	9.97	10.6	11.2	**13.9**	**15.2**	13.0	10.0
Major depth	**2.80**	**5.31**	5.94	7.06	7.54	5.26	**3.28**	**3.02**	5.37	6.76
Minor width	**4.24**	**4.80**	6.92	8.51	8.10	6.14	**3.96**	**4.34**	6.17	8.27
Minor depth	**5.38**	**5.03**	4.63	3.87	4.01	5.31	**5.71**	**5.15**	4.74	4.59

Further analysis of the binding specificity and experimental biochemical data shed some light on the nature of the cooperativity. The interaction of ATF-2 with the consensus site TGAC (Figure 1) involved specific HBs with bases and electrostatic interactions with the DNA backbone, with an Asn344 side-chain HB with T_-99_ and G_-98_ (C of the complementary strand), and Arg352 HB with C (G of the complementary strand). On the other hand, c-Jun interacts with (non-consensus) DNA backbone without any specific HB with the bases. Interestingly two other similar structures involving c-Jun (1JNM and 2H7H) were found to have no HBs with bases either, suggesting that indeed binding of c-Jun could be of lower DNA sequence stringency compared to ATF-2. Combined, these results suggest that ATF-2/c-Jun binding orientation and DNA conformational change were dominated by the requirement to selectively favor IRF-3 binding because IRF-3a and c-Jun share two nucleotides. This also explains the previous experimental observation that in the absence of IRF-3, c-Jun/ATF-2 were able to bind their respective sites even when the order of the two sites was reversed [32]. However, when IRF-3 was present, the ternary complex was formed only when the two sites had the wild type sequence. Reversing the order of the DNA binding sites for ATF-2 and c-Jun will put the ATF-2 binding sequence next to the IRF site, hampering native IRF-3 binding.

### Factors dictating binding specificity and cooperativity in the 1t2k complex

**Figure 4:**
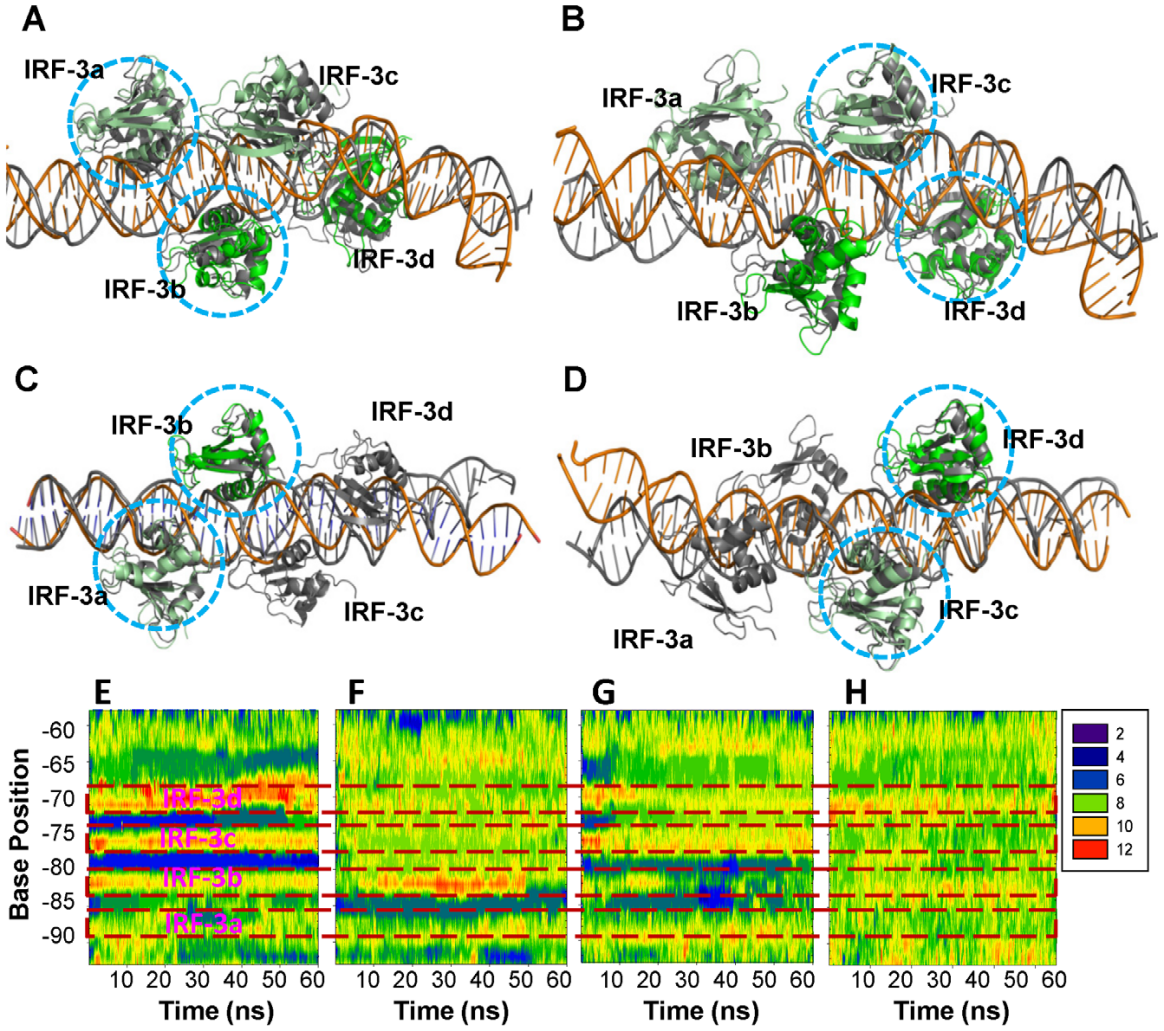
Conformational changes of various structures derived from 2O6G upon MD simulations. (A–D) Superposition of averaged structures over the last 10 ns trajectories for the 2O6G full complex (A and B), the DNA/IRF-3a/IRF-3b (C), and DNA/IRF-3c/IRF-3d (D) complex, respectively. Structural motifs used for superposition are highlighted with circles. Crystal structure is shown in gray. Coloring code as in Figure 1. (E–H) DNA groove parameter changes over the 60-ns trajectory for the full complex (E), the DNA/IRF-3a/IRF-3b (F), the DNA/IRF-3c/IRF-3d (G), and the DNA alone (H), respectively. DNA regions that correspond to binding sites of proteins were boxed in red and labeled with protein names.

Although the sequence of binding events between ATF-2/c-Jun and IRF-3 dimers is unclear, MD simulations revealed that the effect of dimer binding on the DNA conformation is local and limited. It does not appear that one dimer binding pre-configures the entire adjoining RE for the next dimer binding except the overlapped segments. This is evidenced by the relaxation of DNA conformations following removal of either ATF-2/c-Jun or the IRF-3 dimer. Details of DNA conformational changes upon removal of the proteins are given in Figures 2g–j. In the full complex, the groove parameters were dramatically different from site to site (Figure 2g). Upon removal of IRF-3A and IRF-3B, the minor groove next to the ATF-2/c-Jun binding site immediately became larger (Figure 2h) although it partially recovered later in the trajectory. When ATF-2 and c-Jun were removed, minor groove widths at IRF sites were reasonably retained, and the c-Jun binding site conformation was partially preserved, particularly near the IRF-3A end (Figure 2i), suggesting that IRF-3 binding can keep the DNA in favorable conformation for c-Jun binding. Although the DNA organization seems to be loose which allows very limited protein-protein interactions between the ATF-2/c-Jun and the IRF-3 motifs, modeling a conformation with IRF-3 binding one-bp upstream revealed that there would be extensive steric clashes between IRF-3 and ATF-2 and c-Jun (Figure 3 in Text S1). This clarifies why IRF-3A binds to the non-consensus AAAA site, particularly in the presence of ATF-2/c-Jun, even though a consensus site is available one-bp upstream (GAAA). This result shows that binding site overlap was already maximized. Taken together, this suggests that binding cooperativity is achieved largely via overlapped DNA and via limited protein-protein interactions, as evidenced in Figure 4 in Text S1.

### IRF-3 binding specificities are different at consensus and non-consensus sites

As revealed in crystal structures 2O6G and 2PI0, the apparent conformations of the four IRF-3 (IRF-3A, -3B, -3C and -3D) bound to PRDIII and I, respectively, are very similar and are similarly bound to DNA (Figures 1, 4). Only one protein-protein interaction occurred among the IRFs (between IRF-3A and IRF-3C) (Figure 1 in Text S1). However, interestingly the protein-DNA interactions are distinct: for example, those for IRF-3A and IRF-3C (chains e and g from 2O6G) were more extensive, involving both HBs with bases and electrostatic interactions with DNA backbones (Table 3), while those for IRF-3B and -3D were mainly with the DNA backbone (Table 3). Each monomer interacted similarly with the DNA at the minor groove via conserved residues His40 and Leu42 [11]. The significance of the minor groove interaction by these two residues is that the base pairs involved were the two central pairs of the upstream IRF binding site; that is, the two consecutive IRF-3 proteins shared part of the binding site, with one binding from the major groove side and the other from the minor groove. The differences between sites A/C and B/D with respect to the association with DNA lie in the interactions of IRF-3 at the major groove. Arg78 of IRF-3A and IRF-3C formed 3-center HBs with two consecutive G bases at positions −91 and −90 relative to the transcription initiation site (Figure 3a, c); by interacting with these two G bases, IRF-3A also shared a couple of bps with c-Jun. Arg86 formed HBs with the next two A bases and a C on the complementary chain; Arg81 interacted with the DNA backbone; and interestingly, of the three arginines, only Arg81 was conserved in the IRF-3 family. Ser8 also formed HB with the T that forms a bp with one of the two consecutive As bound to Arg86. In contrast, in IRF-3B, Arg78 interacted with the DNA backbone, Arg81 interacted weakly with a G base without forming a HB, and Arg86 formed a HB with an A base. For IRF-3D, Arg78 formed HB with a T base; Arg81 interacted with DNA backbone while Arg86 was not in close contact with any DNA bases or backbone. These data show that while all IRF-3 proteins were able to form some HBs with DNA bases, and thus render some specificity, the extent of the specificity varied due to differences in HBs. IRF-3A and -3C were more specific and IRF-3B and -3D were less so.

**Table 3:** IRF3 key residue interactions with DNA at the major groove in different crystal structures.

Complex	Residue	IRF-3a	IRF-3b	IRF-3c	IRF-3d/IRF-7d
1T2K/**2O61**	Arg78/T1093	T88(Me) T89(Me)	T81(Me) T82(Me)	**G79(2.81) G78(3.87)**	**T65(Me)**
	Arg81/Arg1096	Backbone	Backbone G83(3.32)	**Backbone**	**Backbone G70(3.56)**
	Arg86/Arg1100	A89(3.81) A90(3.91)	T80(3.49) A81(3.99)	**G75(1.88, 2.87)**	**Backbone A69(4.33)**
	Ser82/Cys	T89(Me)		**C75(3.53)**	
2O6G	Arg78	G88(2.91, 3.01) G88(2.77) G87(2.98)	Backbone (weak)	G78(2.35) G79(2.57)	T70(Me)
	Arg81	Backbone	backbone G83(3.17)	Backbone	backbone
	Arg86	A87(3.01) A87(2.53) T86(3.31)	A81(2.82)	A75(2.82) A76(3.57) T(3.53)	
	Ser82	T87(3.01)			
2PI0	Arg78	G91(2.76) G90(via H_2_O)	backbone	G78(3.21) G77(2.95)	
	Arg81	backbone A89(3.55)	Backbone G83(3.25)	Backbone G78(4.11)	Backbone G70(3.35)
	Arg86/Ala86	A86(2.99) G85(2.99)	A(3.48) A(3.77)		A67N6(3.25) C66N4(3.05)
	Ser82	T87(3.57)	T81(3.01)	C75(3.17)	A68(3.27)
T_(-102)_ AA_(-100)_ ATGAC ATAGG_(-90)_ AAAACTGAAA _(-80)_ GGGAGA AG_(-72)_ (1t2k)					
T_(-102)_ AA_(-100)_ ATGAC ATAGG_(-90)_ GAAACTGAAA _(-80)_ GGGAAA GTGA _(-70)_ AAGTG (2O6G)					
ATAGG_(-90)_ AAAACTGAAA _(-80)_ GGGAGAAGTG _(-70)_ AAAGTG (2PIO)					
TTGAAA_(-80)_ GGGAGAAGTG _(-70)_ AAAGTGGGAA _(-60)_ ATTCCTCTG (2O61)					

The base number shown inside the table omitted the negative sign for simplicity. The numbers in parentheses are the hydrogen bond distances unless the specific atom types were given. Only the contact distances less than 4.5 Å are shown.

The binding patterns for crystal structure 2PI0 [28], which differ by one base pair each in sites A and C and by having a 3 bp spacer instead of 2 between binding sites IRF-3C and -3D, were essentially the same in terms of the general specificity trend (Table 3); that is, interactions for IRF-3A and -3C were more extensive and specific than for IRF-3B or -3D. Alignment of partial structures revealed that both the protein and DNA segment involved in direct contact matched very well between the two structures (Figures 2D, E in Text S1). The only difference is that Leu42 and His40 interacted at the minor groove with two terminal bps instead of the two central ones.

### Dynamics and cooperativity revealed by simulations of DNA/IRF-3 (2O6G/2PI0)

**Figure 5:**
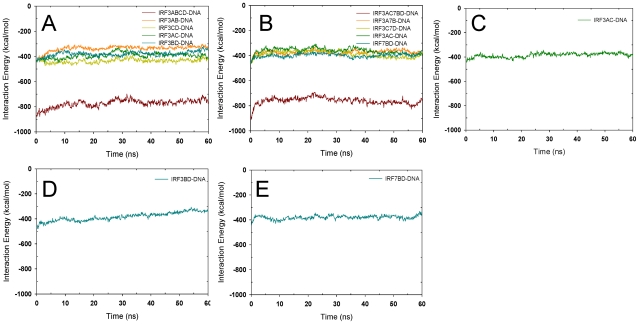
Protein-DNA interaction energies for various complexes derived from the 2O6G crystal structure. (A)–(E) are for the 2O6G full complex, the full complex with IRF-7 at the b and d positions, DNA/IRF-3a/IRF-3c, DNA/IRF-3b/IRF-3d, and DNA/IRF-7b/IRF-7d respectively.

**Figure 6:**
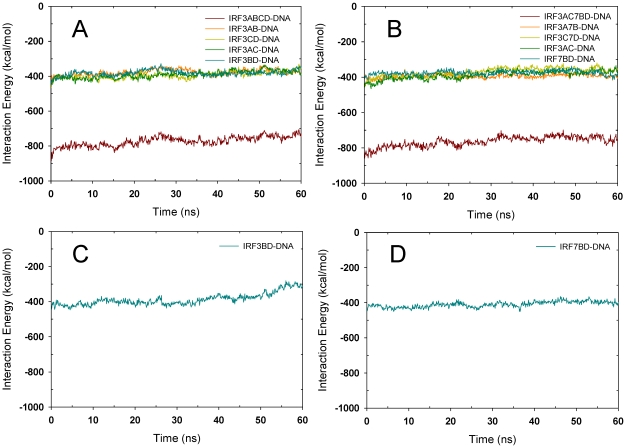
Protein-DNA interaction energies for various complexes derived from the 2PI0 crystal structure. (A)–(D) are for the 2PI0 full complex, the full complex with IRF-7 at the b and d positions, DNA/IRF-3b/IRF-3d, and DNA/IRF-7b/IRF-7d, respectively.

MD simulations were performed on both 2O6G and 2PI0 which are only slightly different in DNA sequence and complex conformation as described earlier. Simulations of the full complex 2O6G revealed that as expected, DNA fluctuation was smaller in the IRF-3 bound region than at the terminal (Figures 4a, b). When only the DNA/IRF-3A/-3B or the DNA/IRF-3C/-3D complex were simulated, DNA conformation at the IRF-3 bound region was again relatively conserved (Figures 4c, d); however, the DNA region now deprived of IRF-3 relaxed and deviated from the starting structure. The complexes with the motif combinations DNA/IRF-3A/IRF-3C and DNA/IRF-3B/IRF-3D were also simulated to evaluate whether binding of a dimer on the same DNA side (AC or BD) would be different from that on opposite sides (AB, or CD) since experimentally, cooperative binding of IRF-3 dimers exists only when both PRDI and PRDIII sites are present [34]. The results from these simulations were similar in terms of DNA conformational dynamics (data not shown). Since binding of two IRF-3 molecules at sites A and C and two IRF-7 molecules at sites B and D is the functionally relevant mode, the DNA/IRF-3A,-3C/IRF-7B,-7D was modeled and simulated as well. While the global conformational changes of the full complex were similar to those of the DNA/IRF-3A/-3B/-3C/-3D simulation results, the protein-DNA interaction profile did reveal some differences. In the DNA/IRF-3ABCD complex, the IRF-3 interaction energies with DNA were more spread while for the DNA/IRF-3AC-7BD complex these interactions were closer to each other (Figures 5A, B), although this feature is not obvious for the 2PI0 complex simulation (Figures 6A, B). Furthermore, the interaction energy for IRF-3BD with DNA was less favorable than that of the IRF-7BD for both 2O6G and 2PI0 complexes (Figures 5C, D, E and 6C, D). Other interaction energies were also calculated and presented in Figure 5 in Text S1. These results indicate that positions B and D prefer IRF-7 while A and C favor IRF-3.

Analysis of the DNA groove parameters confirmed the limited impact of one IRF binding on the other. In the 2O6G complex, there was significant minor groove narrowing between binding sites (Figure 4e). After removal of IRF-3C and IRF-3D or IRF-3A and IRF-3B, these structural features completely disappeared in the region where IRF-3 was removed, whereas the IRF-3 bound region still remained close to that of the crystal structure (Figures 4f, g). In the DNA/IRF-3C/-3D complex, the minor groove for the spacer region between sites B and C remained narrow, suggesting that much of the binding site for IRF-3B was in a ‘ready’ state because the sites overlap (Figure 4g). Comparison of DNA parameters between the full and partial complexes shows that there is some impact on the overall DNA conformation when the two dimers were bound together (Figures 4e, f and g). In the absence of proteins, the groove parameters were characteristic of free DNA (Figure 4h).

### The structural basis for the cooperativity and preferences of the IRF-3/IRF-7 proteins for specific DNA sites

Similar to the 1T2K complex, the simulations of 2O6G did not show that the binding of one IRF-3 dimer was able to keep the neighboring DNA full sites in the crystal structure conformation. However, it did show that the DNA conformations in the IRF-3 bound region were well retained. Because the binding sites for the two IRF-3 dimers (or monomers) overlap significantly, cooperativity can take place through a pre-organization of the overlapped DNA concomitant with the binding of one dimer. The DNA conformation in the full complex differed from that of the DNA/IRF-3 dimer, suggesting cooperative strengthening of the interaction of each with the DNA.

**Figure 7:**
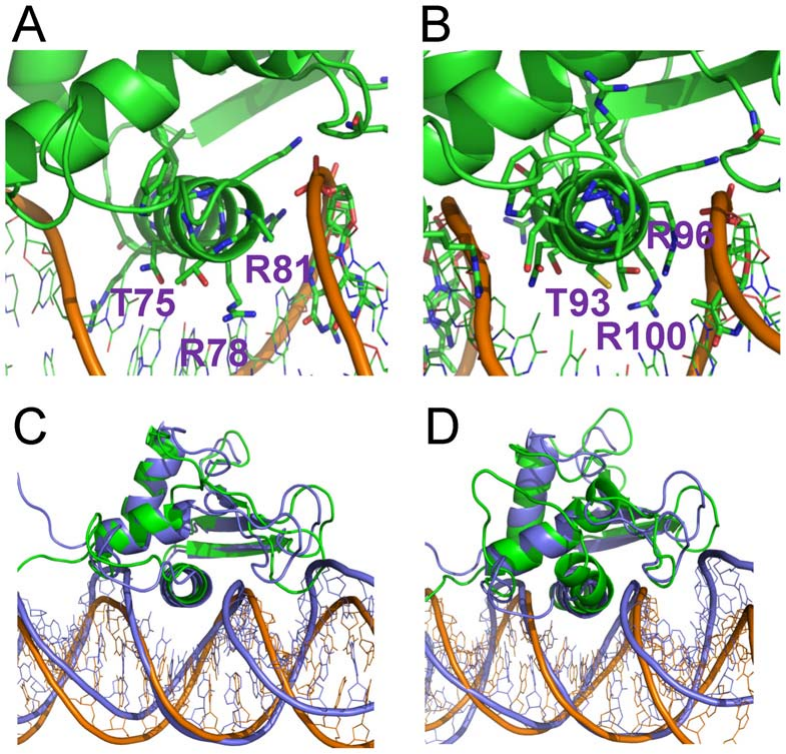
Different DNA binding and interactions of IRF-3 and IRF-7 at two binding sites. (A) and (B) Average structures of DNA/IRF-3 and DNA/IRF-7 motifs at the binding sites c and b, respectively, from the 2PI0 complex simulations trajectories for the last 10 ns. Residues T93 and R96 in IRF-7 corresponding to R78 and R81 are shown. (C) and (D) Superposition of the averaged structures IRF-3 (C) and IRF-7 (D) (in green and orange) bound at binding site b onto the crystal structure (in purple), showing the different binding tightness of IRF-3 and IRF-7.

Above, we showed that the interactions of Arg78 were different at the four IRF binding sites, with sites A and C similar to each other, and different from B and D (Table 3, Figure 3). The main reason why Arg78 oriented differently at sites B and D relates to the T base preceding the consensus sequence (Figure 1). Due to the protruding methyl group from the T, Arg78 could not form stable HB with the G within the binding sites and was forced to turn away (Figures 3b, d). When IRF-7 was bound at these two positions, such steric conflict did not exist, fitting snugly at the sites. Figure 7 shows that the binding of IRF-7 at the B site was different from that of IRF-3 at the same site because the residue at the Arg78 position was Thr93 which has a shorter side chain and thus able to make hydrophobic interactions with the otherwise unfavorable methyl group of T (Figures 7a, b). As a result, IRF-7 binds DNA more tightly at sites B or D than IRF-3 (Figures 7c, d).

### Analysis of the interactions in the 2o61 (DNA/IRF-3C/IRF-7D/p50/RelA) structure

In the 2o61 crystal structure, interactions between IRF-3C and IRF-7D are sparse, with only one HB between Arg60 of IRF-3C and Ser125 of IRF-7D, which is the C-terminal residue (Figure 1 in Text S1). Interactions between p50 and RelA are extensive (Figure 6 in Text S1). Analysis of the protein-DNA interactions again revealed an interesting phenomenon. IRF-3C interacts with DNA in a pattern similar to what was described for the 2O6G complex. However, the IRF-7D interaction is more extensive and specific than IRF-3B and IRF-3D in 2O6G (Table 3). Thr, which replaced the IRF-3 Arg78, did not need to bend or re-orient to avoid the steric conflict with the underneath T base. Instead, it made van der Waals/hydrophobic contact through the methyl group.

### Dynamics of the DNA/IRF-3C/IRF-7D/p50/RelA complex

**Figure 8:**
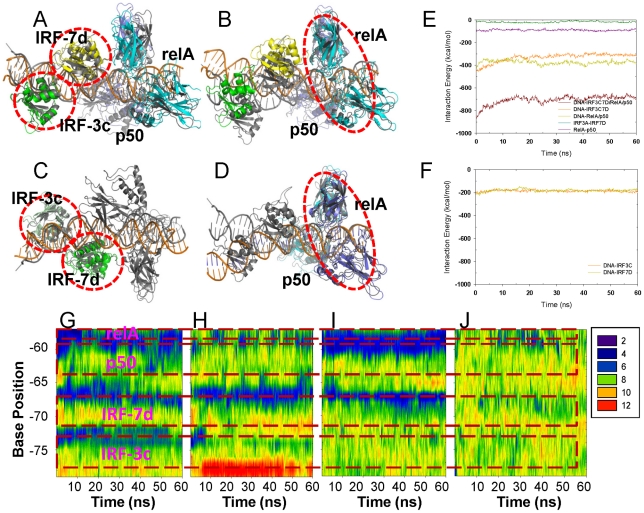
Conformational and protein-DNA interaction properties from the 2O6I complexes simulations. Superimposition of average structures over the last 10 ns of the trajectories for the full complex (A–B), DNA/IRF-3c/IRF-7d (C), and DNA/p50/RelA (D) on to the crystal structure. The superimposed motifs were highlighted in red circles. Protein-DNA interactions for the full complex (E), DNA/IRF-3c (F) and DNA/IRF-7d (F). (G–J) are the DNA minor groove parameters for the full complex, DNA/IRF3c/IRF-7d, DNA/p53/RelA, and DNA respectively.

In the full complex simulation, both the local conformations and the overall structure were retained relatively well compared with the 1t2k complex, although the conformational difference from the crystal structure was still noticeable (Figures 8a, b): the simulations of DNA/IRF-3C/IRF-7D and DNA/p50/RelA complexes show that DNA conformations were minimally perturbed at the binding sites (Figures 8c, d) while the overall structures significantly drifted from the crystal conformation, which was expected. This result illustrated again that the DNA conformation fluctuation and the relatively large movement between the segments was the consequence of the sparseness of protein-protein interactions on different DNA segments.

Details of the protein-DNA interaction energies are presented in Figures 8e, f. A few interesting observations can be noted: 1) The interaction energies between the DNA and p50/RelA were very similar in the full complex and in the p50/RelA-DNA motif, indicating stable interactions for this association (data not shown); 2) the interaction energies for IRF-3 and IRF-7 with DNA were very similar to each other (Figure 8f), suggesting that the IRF-7 binding at the D (and the B) site was more favorable than the IRF-3 binding at the same sites. This observation is consistent with the simulations results of the 2O6G and 2PI0 full complexes with IRF-7 bound at the B and D sites. DNA groove parameter analysis also revealed limited yet observable DNA conformational impact by protein binding at the neighboring site (Figures 8g–j). When the p50/RelA dimer was removed from the complex, the minor groove width downstream of IRF-7D did not change significantly (Figure 8h). However, the conformation in the IRF-3C and IRF-7D bound region did not maintain well in the crystal structure, suggesting that IRF binding was not as tight in the absence of p50/RelA. On the other hand, when IRF-3C and IRF-7D were removed, the p50 and RelA bound portion retained well the crystal structure conformation, highlighting the stability of this protein-DNA motif (Figure 8i). In this case, the DNA conformation for the IRF-7D binding site was also similar to the crystal structure, confirming the impact of p50/RelA binding on the DNA conformation at the IRF-7 site.

## Discussion

### High flexibility is characteristic of this enhanceosome

The combinatorial assembly mechanism of TFs in the enhanceosome is of paramount importance. Even for the small IFN-β enhanceosome, despite considerable cell biology, biophysics, and structural characterization work, it is still unclear how the three modules are selectively recognized and come together to lead to transcription initiation. From the functional standpoint, the IFN-β enhancesome complex can be roughly divided into three modules: ATF-2/c-Jun, IRF, and p50/RelA sites listed from upstream to downstream (Figure 1). While we have shown that packing has reached maximum tightness, the complexes demonstrated high flexibility, higher than typically observed in protein-DNA complexes where there exist extensive protein-protein interactions. DNA can be very flexible, capable of forming sharply looped DNA-protein complexes [35]. However, complexes where two proteins bind shoulder to shoulder on a DNA segment with high specificity and extensive protein-protein interactions, allow very limited DNA fluctuations. For example, the complex of the p53 tetramer with DNA presents very limited DNA conformational change or DNA bending, with a maximum of 30 degrees of curvature only when the DNA sequence is optimized [36], which is evidenced in low resolution experiments. Such dynamic properties can be demonstrated through MD simulations, and is not always captured in crystal structures possibly due to crystal effects.

### Overlap of REs leads to cooperative, thus selective TF binding on enhancer DNA

The salient feature that the IFN-β enhanceosome harbors few protein-protein interactions suggests that assembly cooperativity could stem from DNA conformational changes following protein binding; that is, TF binding-induced conformational changes may propagate along the DNA, pre-configure neighboring REs for optimal binding by a second TF, and this could be a key factor in RE recognition. Yet, our results show that the direct effect on DNA conformation by binding of a TF dimer is limited to only the neighboring sites. This is supported by our simulation results that removing a protein molecule from the complexes will cause the DNA conformation to drift away from that in the crystal structure, with only a few bps next to the binding sites reasonably retaining the crystal conformation. Thus, instead of long range DNA allosteric effects, our results suggest that overlap of binding sites is the mechanism of enhanceosome binding cooperativity, between ATF-2/c-Jun and IRF-3A, among IRFs, and between IRFs and p50/RelA proteins. Overlap of binding sites is reasonable and likely to be a broadly utilized enhanceosome mechanism. Constructs with different overlaps of REs and abolished protein-protein interactions may help in delineating the impact of these conformational factors on transcription.

### Dimer binding and interactions between different dimers

Hetero-dimerization of TFs is widely recognized and known to be important for binding specificity and consequently function [37]. Experimental data show that pairs of the enhanceosome TFs are often expressed together. For example, the RelA/p50 and RelB/p50 data suggest that they are synthesized at the same time, and are found in complex with p100 in the nucleus [38], [39] and bind DNA first [40]. The question is why unique combinations of ATF-2/c-Jun, IRF-3/IRF-7 and p50/p65?

NF-kB (p50/RelA) is a ubiquitous eukaryotic TF which plays critical roles in transcription of numerous genes [41] and is often modified [42]. Like the ATF-2/c-Jun dimer, it is present in most cells and involved in many biological processes including proliferation, differentiation, and apoptosis [43]–[45]. p50/RelA dimerization is important for transcription. Since the binding specificity is high and the dimerization interface is stable, the binding of this motif is expected to contribute significantly to the stability of the enhanceosome. Interestingly, when the spacer between p50/RelA and IRF-7D changes from 2 to 3 nucleotides the transcriptional activity is only slightly affected. Because the two binding sites still overlap by 3–4 bps with the 3-bp spacer, it is understandable that cooperativity, and thus function, is only minimally changed.

ATF-2 and c-Jun belong to a super-family of TFs that share the basic-region Leucine-zipper motif but have different DNA binding specificities. The ATF-2/c-Jun heterodimer is more populated and binds DNA tighter than either homodimer [46]. c-Jun by itself recognizes the so-called AP-1/TRE site with the symmetrical sequence TGACTCA while ATF-2 recognizes the ATF/CRD consensus site TGACGTCA, which is also symmetric [47]. The difference is in one bp. This difference may suggest that c-Jun dimer binding is not as specific as the ATF-2 since it binds to smaller sites (TGA) while ATF-2 needs two TGAC sites. Combining previous work which shows that the assembly of ATF-2/c-Jun/IRF-3 complex occurs only when the DNA sites were in the ‘right’ order [32] and our simulation results, it is likely that the non-consensus site is only for c-Jun binding since structural analysis demonstrates that it has few specific interactions with the DNA. Thus, nature has designed the DNA sequence and the ATF-2/c-Jun dimer for optimized binding specificity of each TF and cooperativity between neighboring partners.

IRF-3 activation requires dimerization through phosphorylation [48] which appears controlled by acetylation [49]. However, the IRF-3 dimerization benefit is not obvious, as there is almost no interaction between the DNA binding domains either on the same or opposite sides of the DNA. In addition, it seems that IRF-3 at sites B and D can be easily replaced by IRF-7, since IRF-7 binding at these two positions is more stable than IRF-3 binding. Therefore, the initial binding mode of dimeric IRF-3 (same- or opposite-side of the DNA) may not be as important as previously thought and IRF-3/IRF-7 dimerization should also be favorable. Because the binding of the IRF DNA binding domain was weak when the other proteins were absent [29], dimerization may allow concurrent binding, which enhances not only the binding affinity, but also the specificity, excluding other TFs from binding to the same sites. Interestingly, the IRF-5/IRF-7 dimer is a repressor of IFN genes [50]. Further study is needed to gain insight into the structural basis of this difference between IRF-3 and IRF-5 binding.

### Environment affects TF-RE specificity

Assembly of a unique enhanceosome depends on factors such as the chromatin state, i.e., whether the enhancer is available, the TFs concentration and post-translational modification states, and TFs affinity to their respective REs [1]–[3], [51], [52]. Specificity also relates to binding of partners (and cofactors) since allostery and structural reorganization are always involved in conformational perturbation during binding [53]. A recent analysis of 8mer REs [54] suggested that while each TF has sequence preferences, just about half of the TFs bind to distinct DNA motifs. TFs from even the same family may show large differences in affinity and site preference [2], [3], [9]. Related to our case, IRF-4 and IRF-5 both bind strongly to DNA containing CGAAAC segments but weakly to TGAAAG and CGAGAC; and specifically, IRF-3 prefers sites A and C while IRF-7 has higher affinity toward B and D. Although there is distinct sequence preference [55] and some correlation between binding affinity and specificity [56], RE sequences are not the only factor that determines what will bind. As shown in table 3, various binding patterns were observed in complexes with similarities at specific positions. For example, binding patterns of Arg78 and Arg86 were different in two crystal structures (PDB 1T2K and 2PI0) at identical non-consensus sites, while other residues including Arg81, Ser82 and Ala83 interacted with DNA in almost the same way. In one case (2PI0), both arginines formed HB with respective bases, while in the other (1T2K) Arg78 only interacted with the methyl groups of two thymines. One of the major differences between the two complexes is that in 1T2K, ATF-2/c-Jun dimer bound upstream of the IRF-3A, which forced Arg78 to point inward and to interact with bases within its own binding sites. As a result, Arg86 adjusted its interactions as well. Similarly, although IRF-3 binding at sites B and D was not optimal relative to IRF-7, it was able to bind at these sites with adjusted orientations, resulting in transcription upon viral infection. Of interest, TFs from the same family that share similar DNA binding domains often have different functions [57]. These could reflect altered cofactor binding sites, the outcome of RE-induced allosteric propagation.

**Figure 9:**
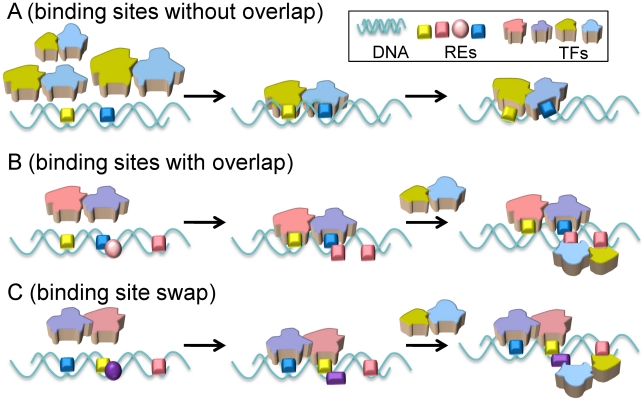
Schematic illustration of the role of RE organization in selective TF binding. (A) A certain size spacer between the two REs excludes the binding of two proteins that are too small (lacking favorable contacts), or too large (will have steric clash). (B) Two proteins bind partially overlapped REs on different DNA faces. Binding of the first protein reconfigures the overlapped DNA conformation (opposite side), leading to more favorable binding of the second. (C) Switching the order of neighboring REs impacts the overlapped binding site, disfavoring binding of the corresponding protein.

To conclude, our work emphasizes the crucial, yet largely overlooked role of the organization of successive REs along regulatory DNA stretches, such as enhancers and promoters, in specifying TF binding selectivity. To date, efforts have largely focused on analysis of binding sites and derivation of consensus sequences. Yet, the order of REs and the spacers between consecutive REs can also play a critical role (Figure 9). Spacer sizes determine the TF shape and dimensions: TFs which are too large or too small are disfavored due to either steric effects (Figure 9a) or lack of interactions with the adjoining TFs (Figure 9a). Overlapping REs (Figure 9b) can function via *cooperative* effects through the binding of TFs to complementary bases, excluding disfavored TFs or enhancing those with relatively low affinity. We propose that overlap of REs is a general mechanism in enhanceosome assembly, beyond the IFN-β. Finally, the order of the binding sites can also be expected to have a functional significance, with a reversed order (Figure 9c) functioning as a repressor. It will be interesting to test the role of spacers by *in vivo* experiments, where other TFs are also present. Genome searches for identical binding sites but with reversed order are expected to uncover additional occurrences of such a functional mechanism which could be tested experimentally. Combined with current experimental data, our results lead us to propose key factors in RE selectivity and functional TF assembly: exposed (i.e. not covered by nucleosomes) enhancer DNA, available for TF binding; RE sequence and order; the length (positive or negative) of spacers between REs; the TFs concentration and post-translational modification states; and proteins and DNA conformational ensembles. Here, our study emphasizes the key role of cooperativity in making the REs a functionally unique gene regulation site. RE organization along the DNA and the intervening spacers play a key role in selective combinatorial assembly, and as such, in the regulation of gene expression.

## Materials and Methods

### MD simulation protocol

MD simulations were performed on four partial enhanceosome crystal structures and their components [28]–[30]. The composition of each simulation is listed in table 1. Each system was solvated with a TIP3P water box [58] with a margin of at least 10 Å from any edge of the box to any protein or DNA atom. Solvent molecules within 1.6 Å of the DNA or within 2.5 Å of the protein were removed. The systems were then neutralized by adding sodium ions. The resulting systems were subjected to a series of minimizations and equilibrations using the CHARMM program (academic version) [59], [60] and the CHARMM 22 and 27 force field for the protein [61] and nucleic acid [62], [63], respectively. The production MD simulations were performed at temperatures of 300 degrees Kelvin using the NAMD program [64] and the CHARMM force field. Periodic boundary conditions were applied and the non-bonded lists were updated every 20 steps. The NPT ensemble [65] was applied and the pressure kept at 1 atom using Langevin-Nose-Hoover coupling [66]. SHAKE constraints [67] on all hydrogen atoms and a time step of 2 fs and a nonbonded cutoff of 14 Å with force shift algorithm were used in the trajectory production. Electrostatic interactions were treated with particle mesh Ewald algorithm [68], [69]. The sizes of the systems were about 110,000 atoms and the duration for each simulation was 60 ns.

### Modeling of enhanceosome complexes

Two complexes were modeled that constituted the DNA IRF-3ac/IRF-7bd with both the 2O6G and 2PI0 templates. In addition, because some of the residues were missing in the crystal structure of 2PI0, IRF-3 structure at position B was used to model IRF-3 at positions A and D. These complexes were constructed by superimposing the backbone of IRF-3 or IRF-7 onto the proteins that were originally there. The systems were minimized for 2000 steps with the ABNR algorithm. The obtained structures were then solvated and further minimized as described in the previous procedures. DNA parameters were calculated with the CURVES program [70], [71].

## Supporting Information

Text S1 Supplemental information file.
(DOC)
