# Supplementary material for: The Role of Response Elements Organization in Transcription Factor Selectivity: The IFN-β Enhanceosome Example
Source: PLoS Comput Biol. 2011 Jun 16;7(6):e1002077. doi: 10.1371/journal.pcbi.1002077 (PMC3116919; doi:10.1371/journal.pcbi.1002077)
Supplement: Text S1 — Supplemental information file. (DOC) [file pcbi.1002077.s001.doc]

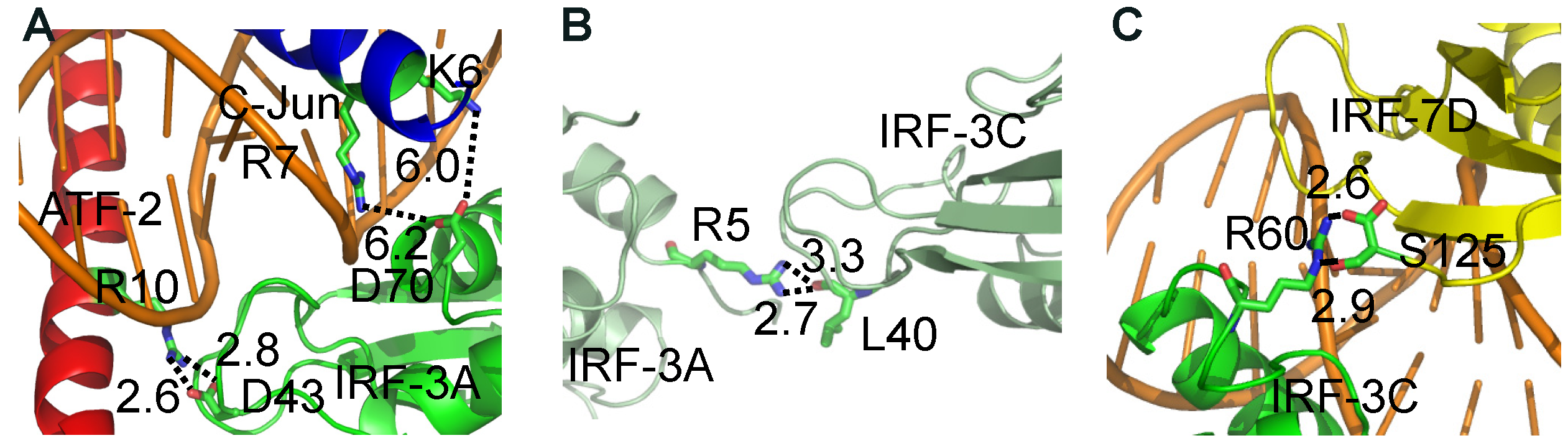
Fig 1. Protein-protein interactions present in the crystal structures 1t2k (A), 2o6g (B), and 2o61 (C). The interactions between p50 and relA and between ATF-2 and c-Jun at the dimerization interfaces are not excluded.


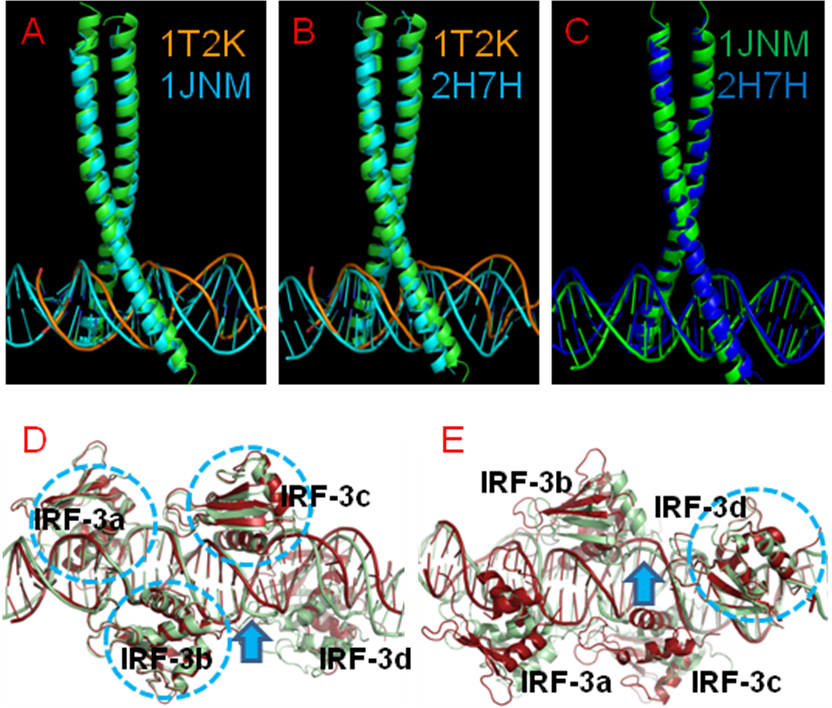
Fig 2. Comparison of crystal structures to illustrate the impact of protein binding on DNA conformations. (A) and (B) superposition of two basic-region Leucine-zipper protein complexes (1jnm and 2h7h) onto 1t2k, showing the huge difference in DNA conformations. (C) Superimposition of 1jnm and 2h7h, revealing similar conformations. Superimposition of structures 2o6g (green) and 2pio (red) by the IRF-3a, -3b and -3c (D) and by IRF-3d (E). The difference between 2o6g and 2pi0 is that 2o6g has an extra bp between binding sites for IRF-3c and IRF-3d versus 2pi0. The alignment shows that the extra bp conformation is similar to an “outlier”, possibly due to the loss of IRF-3b and IRF-3d interactions because of the rotation.

Fig 3. Modeling IRF-3A binding one bp upstream of its cognate site in the 1t2k complex. (A) Crystal structure conformation. (B) Modeled conformation. Red arrow shows the direction of the shift of the binding site and green arrow shows the steric clash between IRF-3a and ATF-2/c-Jun dimer. Modeling was performed through the superimposition of the 5-bp DNA segments that cover the binding sites.


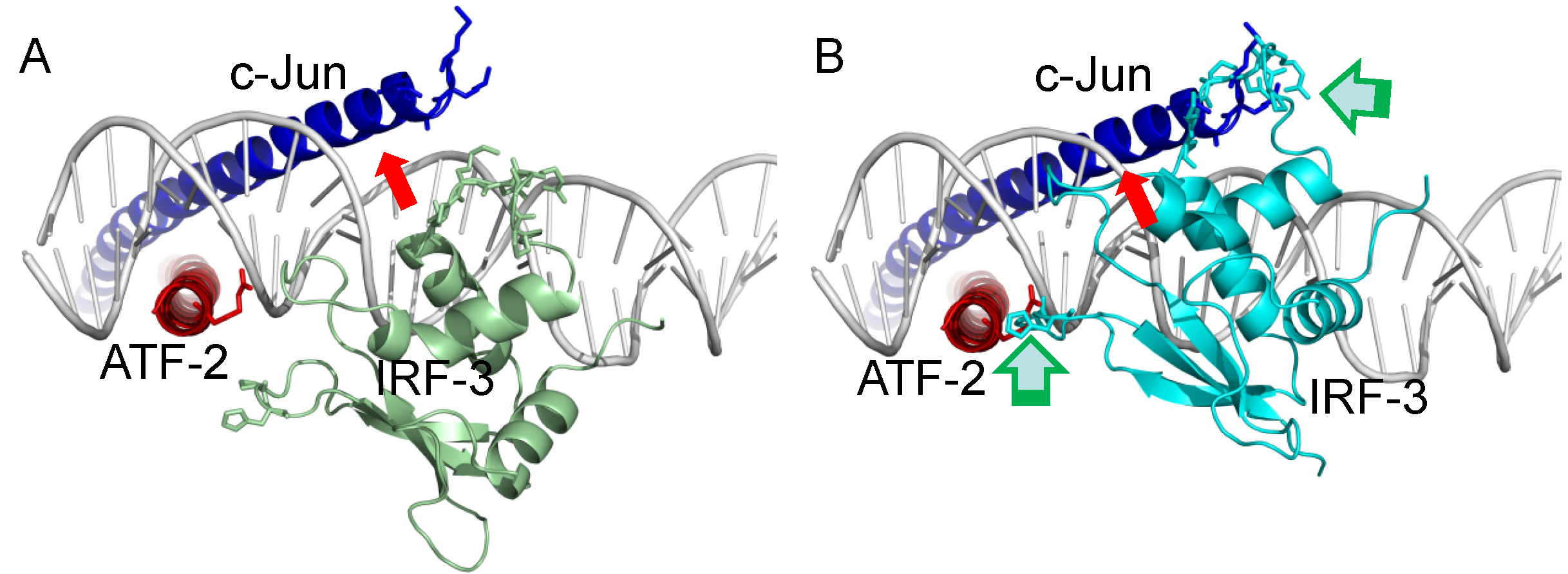


Fig 4. Interaction energies for various interacting partners in different complexes derived from 1T2K. (A) The full complex. (B) The DNA/ATF-2/c-Jun complex. (C) The DNA/IRF-3a/IRF-3b complex. (D) ATF-2/c-Jun complex. (E) and (F) the selected distances between interacting proteins.


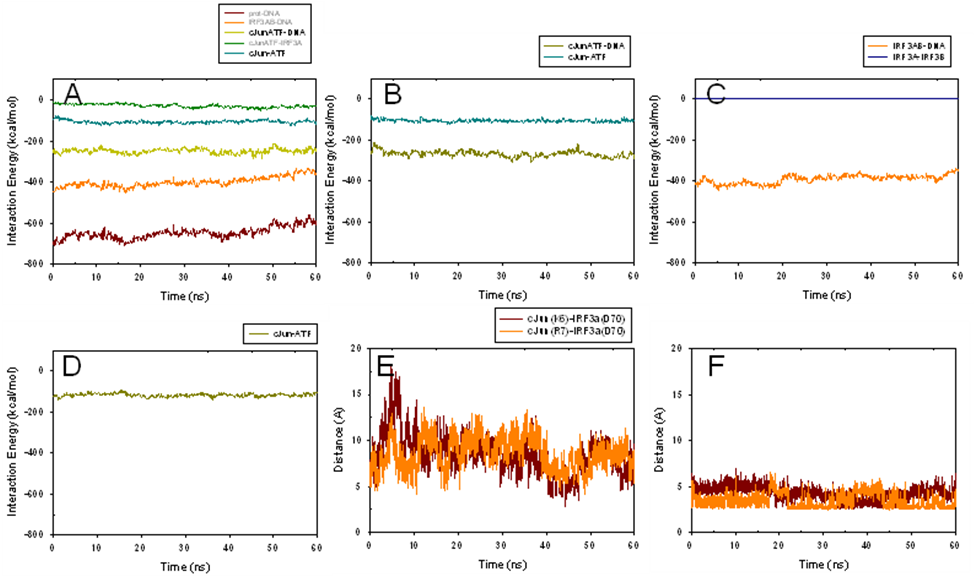


Fig 5. Interaction energies for various interacting partners in different complexes derived from the 2O6G. (A) The full complex. (B) The full complex in which sites B and D were replaced by IRF-7. (C) DNA/IRF-3a/IRF-3b complex. (D) DNA/IRF-3c/IRF-3d complex. (E) DNA/IRF-3a/IRF-3c complex. (F) DNA/IRF-3b/IRF-3d complex. (G) DNA/IRF-7b/IRF-7d complex.


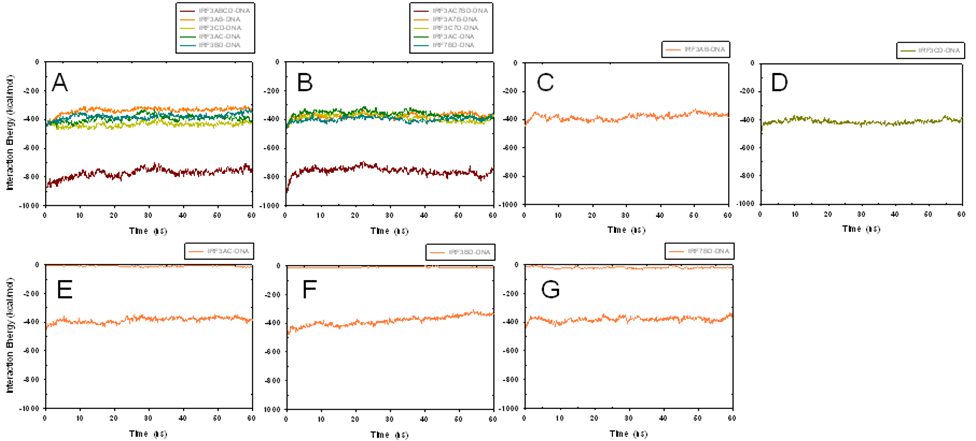


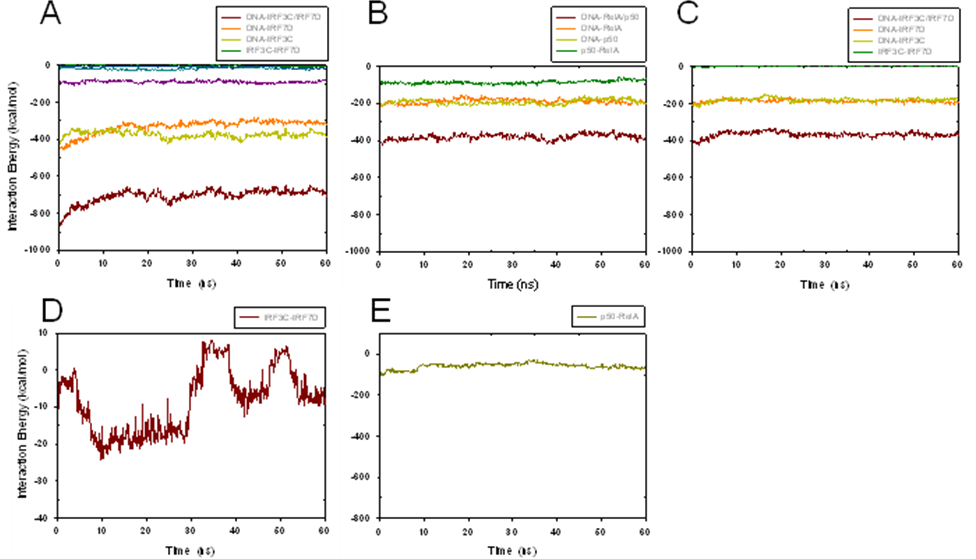
Fig 6. Interaction energies for various interacting partners in different complexes derived from the 2O6I. (A) The full complex. (B) DNA/p50/RelA complex. (D) DNA/IRF-3c/IRF-7d complex. (E) IRF-3c/IRF-7d complex. (F) p50/RelA complex.
